# *PLOS Biology* 2017 Reviewer and Editorial Board Thank You

**DOI:** 10.1371/journal.pbio.2006030

**Published:** 2018-03-19

PLOS and the *PLOS Biology* team would like to express our appreciation for our academic editors, guest editors, and reviewers who contributed to the peer-review process this past year. We are indebted to our volunteers who generously give their time and expertise to thoroughly review research and advance Open Access. In 2017, *PLOS Biology* received the help of 178 Editorial Board members and 131 guest editors to curate 2,743 submissions. Along with the participation of 1,481 reviewers, we were able to publish 160 articles with meaningful and impactful results.

The names of our 2017 editors that handled submitted manuscripts appear in the Supporting Information as S1 Editor List and as S1 Guest Editor List. Our reviewers appear in the Supporting Information as S1 Reviewer List. With genuine gratitude, we thank you all for your dedicated support for *PLOS Biology* and our efforts to promote Open Science, thereby contributing to the scientific community as a whole. Thank you all.

## Supporting information

S1 Editor List Alphabetical list of 2017 *PLOS Biology* Editorial Board Members. (PDF)

S1 Guest Editor List Alphabetical list of 2017 *PLOS Biology* Guest Editors. (PDF)

S1 Reviewer List Alphabetical list of 2017 *PLOS Biology* peer reviewers. (PDF)
