# Supplementary material for: PLOS Biology 2017 Reviewer and Editorial Board Thank You
Source: PLoS Biol. 2018 Mar 19;16(3):e2006030. doi: 10.1371/journal.pbio.2006030 (PMC5858763; doi:10.1371/journal.pbio.2006030)
Supplement: S1 Editor List — (PDF) [file pbio.2006030.s001.pdf]

PLOS | BIOLOGY

PLOS Biology would like to thank all those who served on the journal's Editorial Board in 2017:

Anurag Agrawal
Julie Ahringer
Anna Akhmanova
James Ashe
Alberto Bacci
Nathalie Balaban
Anthony Barnosky
Nick Barton
Konrad Basler
Renata Basto
Gillian Bates
Peter Becker
Hugo Bellen
Richard Benton
Lisa Bero
Avinash Bhandoola
Melanie Blokesch
Russell Bonduriansky
Isabelle Boutron
Josh Brickman
James Briscoe
Marianne Bronner
Kenneth Cadwell
Heather Cameron
Judith Campisi
Fernanda Ceriani
Xuemei Chen
Lars Chittka
Heather Christofk
Andrea Cimarelli
Jeff Coller
Isabelle Côté
Jozsef Csicsvari
Matthew Dalva
Richard Daneman
Arjan de Visser
Frans de Waal
Ghislaine Dehaene-Lambertz
Jonathan Demb
Claude Desplan
Ulrich Dirnagl
Andrew Dobson
Xinnian Dong
Joshua Dubnau
Dan Durocher
Raimund Dutzler
Connie Eaves
Bruce Edgar
Jonathan Eisen
Cagla Eroglu
Mark Estelle
Christophe Fraser
Marc Freeman
Tom Freeman
Karunesh Ganguly
Wendy Gilbert
Jeff Gore
Alex Gould
Douglas Green
Ueli Grossniklaus
Bassem Hassan
Anders Hedenström
Joseph Heitman
Daniel Herschlag
Caroline Hill
David Hillis
Gökhan Hotamisligil
Sui Huang
Simon Hughes
Fred Hughson
Laurence D. Hurst
Anna Huttenlocher
Nancy Hynes
Robert Insall
John Ioannidis
Ole Jensen
Pedro Jordano
Gerald Joyce

James Kadonaga
Sophien Kamoun
Scott Keeney
Laurent Keller
Chaitan Khosla
Jonathan Kimmelman
Karla Kirkegaard
Thomas Kirkwood
Katia Koelle
Adam Kohn
Genevieve Konopka
Achim Kramer
Matthias Landgraf
Michael Laub
Raquel Lieberman
Cecilia Lo
Jason Locasale
Michel Loreau
Sally Lowell
Georgina Mace
Laura Machesky
Malcolm Macleod
Harmit Malik
Philippa Marrack
Sophie Martin
Pascal Meier
Tom Misteli
Aaron Mitchell
Nancy Moran
Craig Moritz
Hélène Morlon
Leonie Moyle
Mary Mullins
June Nasrallah
David Nemazee
Eric Nestler
Phillip Newmark
Paula Oliver
Christine Orengo
Christopher Pack
Csaba Pál
Carole Parent
Tanya Paull
David Pellman
Carl Petersen
Gregory A. Petsko
Jon Pines
Hidde L. Ploegh
David Poeppel
Franck Polleux
Sara Rankin
Emma Rawlins
Andrew Read
Roland Regoes
Jeremy Rich
Steven Riley
David Ron
Sarah Rowland-Jones
Matthew Rushworth
Peter Scheiffele
Sandra Schmid
David Schneider
Trina Schroer
Maya Schuldiner
François Schweisguth
Piali Sengupta
Ben Seymour
Mark Siegal
Anita Sil
Mikael Simons
Anne Simonsen
Agata Smogorzewska
Nicole Soranzo
Victor Sourjik
Ann Stock
Kate Storey
Boris Striepen
Tom Sudhof
Bill Sugden
Paul Taghert
Nicolas Tapon
Graham Taylor
Luis Teixeira
Janet Thornton
Rong Tian
Heidi Tissenbaum
Frank Tong

Chris Tyler-Smith
Leslie Ungerleider
Matt van de Rijn
David Vaux
Leslie Vosshall
Eric-Jan Wagenmakers
Matt Waldor
Gary Ward
Amy Weinmann
Mariana Wolfner
Yukiko Yamashita
Phillip Zamore
Robert Zatorre
