# Supplementary material for: PLOS Biology 2017 Reviewer and Editorial Board Thank You
Source: PLoS Biol. 2018 Mar 19;16(3):e2006030. doi: 10.1371/journal.pbio.2006030 (PMC5858763; doi:10.1371/journal.pbio.2006030)
Supplement: S1 Guest Editor List — (PDF) [file pbio.2006030.s002.pdf]

PLOS Biology would like to thank all those who served as Guest Academic Editors in 2017:

Schahram Akbarian
Vania Apkarian
Tim Barraclough
Allan Basbaum
Oded Beja
Niels Birbaumer
Jennifer Bizley
Ran Blekhman
Jeffrey Boore
Seth Bordenstein
Patrice Bourgin
Charles Bourque
Michael Breakspear
Leonid Brown
Stephen Brusatte
Simon Bullock
Kenneth Chien
Ajay Chitnis
Corinna Darian-Smith
Aniruddha Das
Lars Dietrich
Jonathan Dinman
Anna Dornhaus
Ronald Duman
Ben Emery
Charles ffrench-Constant
Brian Finck
Jonathan Flint
Kevin Foster
Angela Friederici
Karl Friston
Fred Gage
Jean-Michel Gaillard
Jorge Galan
Robert Gereau
Ronald Germain
Asif Ghazanfar
Greg Gibson
Deborah Gordon
Bertie Gottgens
Jeremy Green
David Greenstein
Kalanit Grill-Spector
Kun-Liang Guan
Hiroshi Hamada
Bill Harris
Kenneth Harris
Thomas Hawn
Chuan He
Claus Hilgetag
Lora Hooper
Peter Jonas
Sven-Eric Jordt
Maarten Kamermans
David Keays
Eric Kemen
Henry Kennedy
Marm Kilpatrick
David Kleinfeld
Robert Knight
Graham Knott
Andrew Koff
Eugene Koonin
Rohini Kuner
Ulf Landegren
Nick Lane
Simon Levin
Michael Lichten
Daniel Lopez
Jan Löwe
Gergely Lukacs
Fridtjof Lund-Johansen
Joe Lutkenhaus
Claire Marris
Paul Martin
Mark Martindale
Imelda McGonnell
Jessica Metcalf

Irene Miguel-Aliaga
Laura Miller
Samuel Miller
Eric Miska
Lisa Monteggia
Marc Montminy
Arne Mooers
Richard Morris
Coleen Murphy
Philip Murphy
Michael Pack
Kiran Patil
Samraat Pawar
David Penny
Marie-Claude Perreault
Townsend Peterson
Marco Prado
Jason Reed
David Relman
Robert Ricklefs
Callum Roberts
Rafael Sanjuan
Paul Sauseng
Daniel Sheppard
Celeste Simon
Jonathan Simon
Samuel Sober
John Lee Spudich
Gillian Stanfield
Charles Stevens
Xinzhuan Su
Cassidy Sugimoto
Christopher Summerfield
Cliff Tabin
Andrew Tanentzap
Matthew Thomas
Erdal Toprak
Li-Huei Tsai
Mary Tyler
Wim Vanduffel
Jan-Willem Veening
Ashok Venkitaraman
Justus Verhagen
Matt Wachowiak
Henning Walczak
Rachel Whitaker
Mark Williamson
Kenneth Wolfe
Rachel Wong
Kenneth Yamada
Anthony Zador
Hongkui Zeng
Leonard Zon
