# Supplementary material for: PLOS Biology 2017 Reviewer and Editorial Board Thank You
Source: PLoS Biol. 2018 Mar 19;16(3):e2006030. doi: 10.1371/journal.pbio.2006030 (PMC5858763; doi:10.1371/journal.pbio.2006030)
Supplement: S1 Reviewer List — (PDF) [file pbio.2006030.s003.pdf]

PLOS | BIOLOGY

PLOS Biology would like to thank all those who reviewed on behalf of the journal in 2017:

Alejandro Aballay
Elizabeth Ables
Aziz Aboobaker
Arkhat Abzhanov
Nicola Aceto
Igor Adameyko
Jean Adams
Deepa Agashe
Hideki Aihara
James Ainge
Schahram Akbarian
Josh Akey
Tomas Alarcon
Maria P. Alcolea
Aixa Alfonso
Samuel Alizon
Kevin Allen
Uri Alon
Luke Alphey
Doug Altman
Elena Alvarez-Buylla
Jorge Alvarez-Romero
Luis Amaral
Enrique Amaya
Jose Anadon
Dan Andersson
Laura Andreae
Stephane Angers
Kenneth Angielczyk
Alexandre Antonelli
Janis Antonovics
Matthew Apps
Kazuharu Arakawa
Jeff Ardron
Saúl Ares
Maria Argos
Irina Arkhipova
Luc H. Arnal
Heinz Arnheiter
Steven Artandi
Kaveh Ashrafi
Duncan Astle
Sophie Astrof
Frederic Austerlitz
Aurore Avargues-Weber
Bruno Averbeck
Gautam Awatramani
Rava Azeredo da Silveira
Tom Baden
Martha Bagnall
Mitchell Balish
Cissy Ballen
James Bamburg
David Bannerman
Shaowen Bao
Fernando Baquero
Albert-László Barabasi
Pavel Baranov
Christopher Barker
Alexis Barr
Yves Barral
Gregory Barsh
Natasha Barteneva
Brenda Bass
Michael Bassik
Kevin Bath
Hans Bauer
Vera Baumans
Andreas Baumler
Mark Beaumont
Daniel Becker
Morgan Beeby
Marlene Behrmann
Paul Beier
Grant Belgard
Charles Bell
Hugo Bellen
Suliann Ben Hamed

Roger Benson
Anat Ben-Zvi
Til Ole Bergmann
Lisa Bero
Boba Beronja
Andrew Berry
Jody Berry
Edouard Bertrand
Michel Besserve
Elvire Bestion
Sven Bestmann
Andreas Beyer
Jean-Christophe Billeter
Niels Birbaumer
Douglas Bishop
Craig Blackstone
Keith Blackwell
Martin Blaser
Dawn Blitz
Benjamin Blonder
Theodora Bloom
Bruce Blumberg
Margaret Blume-Kohout
Christoph Bock
Ryan Bogdan
Manuel Bohn
Christophe Bonenfant
Leonardo Bonilha
Mathilde Bonnefond
Tjeerd Boonstra
Erie Boorman
Enrica Bordignon
Jose Borghans
Lutz Bornmann
Patrick Bossuyt
Jeffrey Botkin
Emmanuel Boucrot
Chantal Boulanger
Florence Bourgeois
Philip Bourne
Michael Boutros
Yohann Boutte
Jérémy Bouyer
Ingo Braasch
Riccardo Brambilla
Trevor Branch
Sara Branco
Yaniv Brandvain
Robert Braun
Erhard Bremer
Caroline Brennan
Jonathan Brennan
Robert Brewin
James Briscoe
Christopher Brochu
Michael Brockhurst
Timothy Brodribb
Stefan Broer
Sue Broughton
C. Titus Brown
Joel Brown
Robert Brucker
Angela Brueggemann
Jan Brugues
James Buchanan
Dirk Bucher
Nicolas Buchler
Eugen Buehler
Elizabeth Buffalo
Thomas Bugnyar
Davide Bulgarelli
Emanuele Buratti
Steve Burden
Barrington Burnett
Kathleen Burns
Kathryn Bushley
Geraldine Butler
Arthur Butt
Nina Cabezas-Wallscheid
Ken Cadigan
Yu Cai
Colin Camerer
Aaron Camp
Colin Campbell
Barbara Cannon
Simon Capewell
Andrea Cardini
Jonathan Carlson

Kristian Carlson
Rachel Carmody
Tom Carney
L. Roman Carrasco
Yarimar Carrasquillo
Matt Carter
Anne Casper
Araceli G. Castillo
Eero Castrén
Daniel Catenacci
Michael Caterina
Isabella Cattadori
Marc Cavazza
Thomas Cech
Constance Cepko
Benoit Chabot
Rumela Chakrabarti
Grant Challen
Chris Chambers
Navdeep Chandel
Steve Chang
Dai-Yin Chao
Deborah Charlesworth
Mario Chavez
Benjamin Chen
Jack Chen
Xin Chen
Steven Cheng
Steven Chessler
Hongbo Chi
Cheng-Ming Chiang
Natsuko Chiba
Cheng-Ting Chien
Peter Chien
Ajay Chitnis
Peter Chivers
Wonhwa Cho
Edward Chouchani
Lon Chubiz
Dai Chung
Christine Citti
David Civitello
Andrew Clark
Hans Clevers
Thomas Cline
Jean Clobert
Matthew Cobb
J. Mark Cock
Patrice Codogno
Gregory Cogan
Jeremiah Cohen
Michael Cohen
Nick Colegrave
Laura Colgin
Jean-Francois Collet
Catherine Collins
James Collins
Sheila Collins
Marco Colonna
Lucy Colwell
Luca Comai
Maralice Conacci Sorrell
Joanne Conover
Vaughn Cooper
Vanessa Corby-Harris
Heather Cordell
Phil Corlett
Brian Cornwell
Ignasi Cos
Andrew Cossins
Albert Courey
Roy Cox
Carolyn Coyne
Loralyn Cozy
Alister Craig
Trevor Creamer
Sylvia Cremer
Clay Cressler
Gael Cristofari
Lee Cronin
Yuhai Cui
Bruce Cumming
Hermann Cuntz
Gillian Currie
Stephen Curry
Cynthia Czajkowski
Bertrand Daignan-Fornier
Trevor Dale

Bjorn Dalhus
Yang Dan
Jeff Dangl
Nika Danial
Jayne Danska
Aniruddha Das
Matthew Daugherty
Miles Davenport
Steve Davidson
Jonathan Davies
Rebecca Davies
Matt Davis
Alice Davy
Benjamin de Bivort
Rob De Boer
Mario de Bono
Tania de Koning-Ward
L. M. de la Prida
Rob de Ruyter
Joris De Wit
Chris de Zeeuw
Agnes Dechartres
Prescott Deininger
Maria Mar Delgado
Anthony Dell
Daniela Delneri
Marco Demaria
Francesco DeMayo
Benjamin Deneen
Paul Dennis
Alice Denton
Leon Deouell
Terry Derting
Claude Desplan
Mahalia Desruisseaux
Thomas Dever
Ajay Dhaka
Patricia Di Lorenzo
James Di Santo
Kamran Diba
Sven Diederichs
Susanne Diekelmann
Thomas Diekwisch
Steve Diggle
Nai Ding
Shou-Wei Ding
Ulrich Dirnagl
Jochen Ditterich
Susanne Dobler
Andreas Doncic
David Doniger
George Dragoi
Guillaume Drin
Ryan Driskell
Karl Drlica
Julia Drylewicz
Alan Ducatman
Stefan Duerschmid
Gregg Duester
Bernard Dujon
Ronald Duman
Julien Dumont
Kent Duncan
Richard Duncan
Gary Dunny
Madeleine Durbeej
Susan Dutcher
Daniel Dwyer
William Earnshaw
Gerard Eberl
Timothy Ebner
Richard Ebright
Matthias Eder
Patrick Edger
Steve Edgley
Andrew Edwards
Scott Edwards
Amelia Eisch
Arne Ekstrom
Abdel El Manira
Niles Eldredge
Steven Eliades
Eran Elinav
Yechiel Elkabetz
Ulrich Elling
Ronald Ellis
Arne Elofsson
Michael Elowitz

Maurice Elphick
Hana El-Samad
Holger Eltzschig
J. J. Emerson
Ben Emery
Jonas Emsley
Robert Endres
Denis Engemann
David Entenberg
Ian Eperon
Gisela Erf
Marc Erhardt
Harold Erickson
James Erickson
Robert Ernst
Ananias Escalante
Neir Eshel
Michael Espey
Sergio Estay
Mark Estelle
Mark Evers
Andrew Ewald
Paul Ewald
Vanessa Ezenwa
Daniele Fanelli
Stephen Farmer
Victor Faundez
Mel Feany
Juergen Fell
Tommaso Fellin
Wiebke Fenske
Suzanne Fenton
Brock Fenton
Ger Ferbeyre
Thomas Ferenci
Matthew Ferrari
Pier Francesco Ferrari
Paul Ferraro
Douglas Fields
Angela M. Filipe
Sabine Filker
Natosha Finley
Stacey Finley
Leslie Firbank
Gilles Fischer
Jason Fischer
Valentin Fischer
Robert Fisher
Thomas Flatt
Sarah Flatters
Sarel Fleishman
Jonathan Flint
Csaba Foldy
Heide Ford
Frank Förster
Kevin Foster
Marcos Frank
Paul Frankland
James Fraser
Peter Freddolino
Leonard Freedman
Tom Freeman
Michael Freitag
Christian Frezza
Jason Fridley
Itzhak Fried
Karl Friston
Jonathan Fritz
Mark Frye
Ben Fulcher
Odd Stokke Gabrielsen
Julien Gagneur
Jean-Michel Gaillard
Pierre-Henri Gaillard
Jorge Galindo-Villegas
Gordon Gallup
Neeraj Gandhi
Marin Gantner
Vitaly Ganusov
Feng Gao
Olga Garaschuk
Martin Garcia-Castro
Ethan Garner
Paul Garner
Stephen Garnett
Colin Garroway
Anton Gartner
Luis Garza

Jesse Gatlin
Klaus Gawrisch
Neil Gemmell
Ivelin Georgiev
Fanni Gergely
John Gerhart
Ronald Germain
Jennifer Gerton
Alireza Gharabaghi
Asif Ghazanfar
Partho Ghosh
Sankar Ghosh
Deanna Gibson
Mike Gilchrist
Derek Gilroy
Florent Ginhoux
Brian Glancy
Clark Glymour
Holger Goerlitz
Josee Golay
Emma Goldberg
Diego Gomez-Nicola
Paula Gonçalves
Zhiyuan Gong
Fernando Gonzalez-Candelas
Jeffrey Good
Isabel Gordo
Jeff Gore
Toni Gossmann
Friedrich Götz
Jerome Goudet
Fred Gould
Richard Gourse
John Grady
Thomas Graf
Trevor Graham
Dominique Gravel
Dana Graves
Joe Gray
Ann Graybiel
Andrew Greenberg
Joshua Greene
Robert Greene
Eric Greer
Megan Greischar
David Gresham
Michael Griesser
Amy Griffin
Ashleigh Griffin
Gilly Griffin
Julian Griffin
Marcus Grimm
Thilo Gross
Quinn Grundy
Alexandra Grutter
Daniele Guardavaccaro
Ivana Gudelj
Antoine Guisan
Chris Gunter
Wenjun Guo
Neetu Gupta
Sunetra Gupta
Daniel Gurnon
William Gustafson
Rodrigo Gutierrez
David Guttman
David Haak
Michael Habib
S. M. Mansour Haeryfar
Ziad Hafed
Steffen Hage
Stephen Hagen
Matthew Hahn
Gyorgy Hajnoczky
Michael Halassa
Georg Halder
Benedikt Hallgrimsson
Jeff Hammerbacher
Wolfgang Hammerschmidt
Ester Hammond
Satoshi Hanada
Cara Haney
Elsa Hansen
Ewan Harney
Kelley Harris
Kenneth Harris
Nicola Harris
Sarah Hartley

Eric Harvill
Uri Hasson
Ian Hastings
Stavroula Hatzios
Robert Hausinger
Michael Hausser
Biyu He
Congcong He
Zhigang He
Denis Headon
Joan Heath
Stephen Hedges
Rainer Hedrich
Martin Heil
Carl-Philipp Heisenberg
Bryan Heit
Klaas Jan Hellingwerf
Stephen Helliwell
Elizabeth Henaff
Rudi Hendriks
David Hendrixson
Andrew Hendry
Mike Henne
Ryan Hibbs
Masahiko Hibi
Andres Hidalgo
Claus Hilgetag
Caroline Hill
Geoffrey Hill
David Hillis
Tara Hiltke
Deborah Hinton
Lynette Hirschman
Heribert Hirt
Chih-Ming Ho
Wesley Hochachka
Matt Hodgkinson
Paul Hoffman
Patrick Hogan
Edward Holmes
Christopher Honey
Christian Hong
Mark Honigsbaum
Peter Hore
Aaron Hoskins
Gokhan Hotamisligil
Keith Houck
Aolin Hsu
Xindi Hu
Zicheng Hu
David Huang
Yongping Huang
Maite Huarte Martínez
John Huguenard
Thomas Hummel
Mary Hunsicker
Richard Hunter
Laurence Hurst
Arild Husby
Wieland Huttner
Anthony Hyman
Alexander Idnurm
Leszek Ignatowicz
Masamitsu Iino
Auke Ijspeert
Vito Ilacqua
Iliyan Iliev
Hiroshi Imamizu
Jean-Luc Imler
Patrik Inderbitzin
Nicholas Ingolia
Axel Innis
Dragos Inta
Motoyuki Itoh
William Ja
Joseph Jackson
Samuel Jackson
David James
Michalina Janiszewska
Ralf-Peter Jansen
Cecile Janssens
Daniel Jarosz
Paige Jarreau
Martin Jastroch
Vivek Jayaraman
Anja Jensen
Grant Jensen
Holger Jeske

Sebastian Jessberger
Peihua Jiang
Frank Jiggins
Peng Jin
Suk-Won Jin
Xin Jin
Steven Joffe
Brian Johnson
David Jones
Knud Jonsson
James Jontes
Philippe Juin
David Julius
Michael Jurczak
Allen Kaasik
Rebekah Kading
Igor Kagan
C. Ronald Kahn
Thorsten Kahnt
Daniel Kaiser
William Kaiser
Marko Kaksonen
Taku Kambayashi
Gabriele Kaminski
Ryota Kanai
Lawrence Kane
Kunihiko Kaneko
Prasanth Kannanganattu
Jagmeet Kanwal
Katrin Karbstein
Rotem Karni
Natasha Karp
Richard Kaslow
Eleni Katifori
Anne Katten
Yoji Kawano
Yoshihiro Kawaoka
Jeremy Kay
Kendrick Kay
Christoph Kayser
Peter Keightley
Georg Keller
Raymond Keller
Alexander Kellner
Gemma Kelly
David Kelso
Eric Kemen
Gerd Kempermann
Chris Kennedy
Henry Kennedy
Cheryl Kerfeld
Philipp Khaitovich
Baljit Khakh
Walid Khaled
Ankit Khambhati
Kiarash Khosrotehrani
Samira Kiani
Ikuhiro Kida
Michael Kiebler
A. Marm Kilpatrick
Trevor Kilpatrick
Joon Kim
Young-Joon Kim
Matthew Kimber
David Kimelman
Jonathan Kimmelman
Jean-Rémi King
Brooks King-Casas
Justin Kinney
Drew Kiraly
Kenneth Kishida
Roy Kishony
Robert Kittel
P. J. Klasse
Colin Kleanthous
Allon Klein
Ophir Klein
Miriam Klein-Flügge
Peter Kloeden
James Knierim
Chris Knight
Theo Knijnenburg
Mirjam Knoernschild
Graham Knott
Philipp Koellinger
D. Stave Kohtz
Peter Kok
Arash Komeili

Tadeusz Kononowicz
Bonkyoung Koo
Seung-Hoi Koo
Artyom Kopp
Moshe Koppel
Richard Kormelink
Benoit Kornmann
Kirill Korolev
Antonis Koromilas
Tatiana Korotkova
Martin Korte
Andreas Kortenkamp
Sergei Kosakovsky Pond
Margaret Kosek
Christian Kost
Zoe Kourtzi
Eileen Kowler
Yoshimasa Koyama
Claudine Kraft
Richard Kramer
Dwight Kravitz
Sheldon Krimsky
Daniel Kronauer
Susan Krueger
Damian Krysan
Paul Kubes
Christoph Kueffer
Rolf Kuemmerli
Klas Kullanderq
Rohini Kuner
Sheng-Han Kuo
Edo Kussell
Charalambos Kyriacou
Denis Lafontaine
Peter Lakatos
Edmund Lalor
Manoj Lalu
Markus Lambertz
Eckhard Lammert
Kurt Lamour
Christian Lanctôt
Michael Landy
Chris Lane
Brian Langerhans
Vincent Lariviere
Nils-Göran Larsson
Iñigo Lasa
Douglas Lauffenburger
Simon Laughlin
Joshua Lawler
Stanley Lazic
Ryan Lebouf
Terry Lechler
Adrian K. C. Lee
Robert Leech
Min Ae Lee-Kirsch
Jonathan Lefcheck
Lisa Lefferts
Marc Lefranc
Lorenzo Leggio
Daniel Legler
Renaud Legouis
Joseph Lehar
Ben Lehner
Andrew Leifer
Gavin Leighton
Pierre Leopold
Jason Lerch
Jun-Yi Leu
Angela Leung
Colin Lever
Francis Levi
Bruce Levin
Jon Levine
Gary Lewin
Ceri Lewis
Joseph Lewnard
Steven L'Hernault
Daiqin Li
Jennifer Li
Yiming Li
Peter Lichter
Paul Lieberman
Raquel Lieberman
Wen-Hui Lien
Jiandie Lin
Jinxing Lin
Yihan Lin

Xuefeng Ling
Matthew Linkie
Stan Lipkowitz
Joseph Lipsick
Tom Little
J. Troy Littleton
Haoping Liu
Luning Liu
Peng Liu
Nathan Lo
Brent Lockwood
Patricia Lockwood
P'ng Loke
Loren Looger
Jamie Lorimer
Chris Lorson
Ed Louis
Angeliki Louvi
Christopher Lowe
Jian Lu
Kun Ping Lu
Ying Lu
Sheng Luan
Burkhard Ludewig
Julian Lum
Lawrence Lum
Fridtjof Lund-Johansen
Erik Lundquist
Sophia Lunt
Huan Luo
Jia Luo
Minmin Luo
Nicholas Luscombe
Matthias Lutolf
David Lyons
Heikki Lyytinen
Le Ma
Paul Macdonald
Trudy Mackay
Craig MacLean
Alan MacLeod
Malcolm Macleod
Megan MacLeod
Brian MacVicar
Maricel Maffini
Alexander Maier
Ivan Maillard
William Mair
Sergei Makarov
Scott Makeig
Marzia Malcangio
Laurence Maloney
Anna Malovannaya
Christos Mammides
Zacharias Maniadis
Jörg Mansfeld
Salah Mansour
Farshad Mansouri
Riccardo Marioni
David Mark Welch
Florian Markowetz
Michael Marks
Emily Marquez
John Marshall
Judit Marsillach
Robert Martienssen
James Martin
Jennifer Martinez
Susana Martinez-Conde
Marisa Martin-Fernandez
Claudia Martini
Rui Martiniano
Douglas Mashek
Richard Masland
Justin Mason
Juan Mata
Giuseppe Matarese
Iain Mathieson
Michael Matthay
Erik Matthysen
Seema Mattoo
Daniel Matute
Madelon Maurice
Bruce Mayer
Roberto Mayor
Ali Mazaheri
Colleen McClung
Brian McDermott

Jo McEntyre
Patrick McGowan
Elizabeth McGraw
Randy McIntosh
Erin McKiernan
Richard McLaughlin
Gavan McNally
Amelia McNamara
Michael Meaney
Javier Medina
Sarah Medland
Kathryn Medler
Mayank Mehta
Thomas Melia
Joseph Mendelson
Vilas Menon
Keir Menzies
Hugo Merchant
Frederic Mery
Stéphane Mesnage
Christian Metallo
Frederic Meunier
Kate Meyer
Lars Meyer
John Middlebrooks
Vincent Miele
Emmanuel Mignot
George Mikros
Sarah Millar
Jordan Miller
Louis Miller
Peter Miller
Robert Miller
Samuel Miller
Gabriel B. Mindlin
Daniel Minor
Timo Minssen
Christen Mirth
Dan Mishmar
Eric Miska
Aaron Mitchell
Gilles Mithieux
Takashi Miura
Adi Mizrahi
Marek Mlodzik
Edward Mocarski
Naoki Mochizuki
Attila Mocsai
Peter Mohler
Christine Mohr
Michelle Monje
Blaine Mooers
Sunjin Moon
Adrian Moore
Richard Moore
Vamsi Mootha
Camilo Mora
Joao Morais-Cabral
Erin Mordecai
Pierre Morel
Yasuo Mori
Richard Morimoto
Craig Moritz
Kenta Moriwaki
Tim Mosca
Hong Mu
Conrad Mullineaux
Peter Mumby
Marcus Munafo
Andrew Murray
S. Muthuswamy
Keeve Nachman
Cathryn Nagler
Johnathan Napier
Dick Nässel
David Nathanson
Vaidehi Natu
Daniel Neafsey
Benjamin Neale
Dan Needleman
Liz Neeley
Joel Neilson
Marion Nestle
Angel Nevado
Cameron Neylon
Robert Niederman
James Nieh
Rasmus Nielsen

Jakob Nilsson
Krishna Niyogi
Matthew Nolan
Mohamed Noor
Caren Norden
Bennett Novitch
Aleksandr Noy
Tomoyoshi Nozaki
Andre Nussenzweig
Mary O'Connor
Darren Obbard
Melanie Obermeier
Andrew Oberst
Jonas Obleser
Howard Ochman
John O'Doherty
Claire O'Donovan
Michael O'Donovan
Ayodele Odutayo
Amanda Oglesby-Sherrouse
Sam Oh
Benjamin Ohlstein
Naoko Ohtani
Aharon Oren
Anne Osbourn
Henrik Oster
Richard Ostfeld
Marco Ottaviani
Ross Otto
Sarah Otto
Michael Pack
Pamela Padilla
Peter Palese
Mark Pallansch
Satu Palva
Pier Paolo Pandolfi
Jason Papin
Balázs Papp
Peter Parham
Jin Mo Park
Paul Parren
Simon Parson
Raghuveer Parthasarathy
Kiran Patil
Heather Patisaul
Andrea Pauli
Walter Paulus
Samraat Pawar
Jeannet Paz
Graham Peaslee
Amy Pedersen
Marius Peelen
Stuart Peirson
David Penny
Alex Perkins
Josef Perner
Charles Perrings
Jennifer Perry
Franco Pestilli
Christian Petersen
Lyle Petersen
Townsend Peterson
William Petri
Carsten Pfeffer
Suzanne Pfeffer
Albert Phillimore
Uri Pick
Richard Pickersgill
Susan Pierce
Corné Pieterse
Gwenael Piganeau
Alex Pigot
Zachary Pincus
Lukasz Piwek
Massimo Pizzato
Marysia Placzek
Anne Plant
Jean-Claude Platel
David Poeppel
Karen Polizzi
Katie Pollard
Adam Porter
Brad Postle
John Postlethwait
Christopher Potter
Jeffrey Powell
Thomas Pradeu
Nicholson Price

Trevor Price
J. B. Prins
Kathleen Pritchett-Corning
Emmanuel Procyk
Ignacio Provencio
Luigi Puglielli
Yan Qin
Lars Raberg
Dan Rabosky
Gregory Radick
Michael Ragozzino
David Raible
Olivier Raineteau
Rajit Rajappa
Priya Rajasethupathy
Francesco Ramirez
Joseph Ramsey
Nick Ramsey
David Rand
Urvashi Rangan
Antonio Rangel
David Rasmussen
Peter Ratcliffe
Andrew Read
Heather Read
Jenny Read
Peter Reddien
John Reinitz
Diana Reiss
Diego Restrepo
Daniela Rhodes
Vanessa Ribes
Jeremy Rich
Stephen Richards
Robert Richmond
Neale Ridgway
Ingmar Riedel-Kruse
Bruce Riley
Marco Andrea Riva
Rafael Rivera-Bustamante
David Robbe
Edwin Robertson
Matthew Robinson
Sonia Rocha
Christian Rödelsperger
Jon Paul Rodriguez
Francisco Rodriguez-Valera
Adrienne Roeder
Robert Roeder
Zeller Rolf
Melissa Rolls
Beth Roman
Guillermo Romero
Guillaume Romet-Lemonne
Panteleimon Rompolas
Marian Ros
Ilan Rosenshine
Michelle Rosenzwajg
Susanna Rosi
Frederick Ross
Mike Rossner
Francois Rousset
Aurelien Roux
Peter Rowley-Conwy
Daniel Rozen
Mika Rubinov
Marcelo Rubinstein
Assaf Rudich
Adam Runions
Matthew Rushworth
Andy Russell
Pierre Rustin
Gary Ruvkun
Timothy Ryan
Tomas Ryan
Soojin Ryu
Sung Ho Ryu
Andrey Rzhetsky
James Saenz
Julio Saez-Rodriguez
Jon Sakata
Akira Sakurai
Dorothea Salo
Andrew Samuelson
Johan Sandberg
Kristian Sandberg
Joshua Sanes
Babak Sanii

Susanna-Assunta Sansone
George Santangelo
Arvind Santhanakrishnan
Casim Sarkar
Sovan Sarkar
Peter Sarkies
Jennifer Sass
Dara Satterfield
Uwe Sauer
Tatjana Sauka-Spengler
Timothy Saunders
Hervé Sauquet
Pauline Scanlan
Samuel Scarpino
Daniel Schachtman
Rebecca Schaefer
William Schafer
Anne Scheel
René Scheeringa
Dirk-Jan Scheffers
Reinhold Scherer
Christian Schlötterer
Jutta M. Schneider
Gilbert Schoenfelder
Nina Schor
Bianca Schrul
Florian Schubot
Oren Schuldiner
Hinrich Schulenburg
Dirk Schüler
Harald Schulze
Meredith Schuman
Robert Schuurink
Olivier Schwartz
Jean Schwarzbauer
Markus Schwarzlander
Megan Schwarzman
Michael Scofield
Luca Scorrano
Jacob Scott
Jeffrey Segall
Terrence Sejnowski
Anna Selmecki
Emily Sena
Michael Sendtner
Cathal Seoighe
Nenad Sestan
Richard Sever
David Shackelford
Reza Shadmehr
Aaron Shafer
Sharoni Shafir
Diane Shakes
Vikram Shakkottai
Shihab Shamma
Janie Shelton
Noam Shemesh
Katsuhiko Shirahige
Ilya Shmulevich
Saeed Shoaie
Elena Shpak
Xiaokun Shu
Bob Siegerink
Eric Siggia
Stephan Sigrist
Anita Sil
Shai Silberberg
Alcino Silva
Elizabeth Silva
Gregg Silverman
Tim Simcoe
Andrew Simmonds
James Simmons
Benjamin Simons
Alastair Simpson
Eleanor Simpson
Harinder Singh
Navinder Singh
Lea Sistonen
Michail Sitkovsky
Jan Skotheim
Montgomery Slatkin
Katie Slocombe
Stephen Small
Alison Smith
Chris Smith
Dan Smith
David Smith

Kirk Smith
Gerry Smith
Paul Sniegowski
Charles Snowdon
Michael Snyder
Michael Soares
Harry Sokol
Thierry Soldati
David Soll
Sozanne Solmaz
Gina Solomon
Hongjun Song
Alexander Soukas
Nuno Sousa
Francesca Spagnoli
Marc Spehr
Sarah Spiegel
Michael Springer
Charles Springer
Nelson Spruston
John Lee Spudich
Sadeesh Srinathan
Supriya Srinivasan
Jason Stajich
E. Richard Stanley
Tim Stearns
G. Christopher Stecker
Christopher Stefan
Jens Stein
Eirikur Steingrimsson
Wolfgang Stephan
Patrick Stephens
Benjamin Steventon
Randy Stockbridge
Francesca Storici
Dan Stowell
Andrew Straw
Daniel Strech
Daniel Streicker
Ben Strowbridge
Alexander Strunnikov
Yi-Hsien Su
Thomas Sudhof
Cassidy Sugimoto
Scott Summers
Jianjun Sun
Yangang Sun
James Surmeier
Ann Sutherland
Shinsuke Suzuki
Yutaka Suzuki
Nathan Swenson
Michiko Taga
Hong Tang
Marc Tatar
Ann Tate
Cormac T. Taylor
Eric Taylor
John Taylor
Tracy Teal
Jeff Teeters
Luis Teixeira
Aurelio Teleman
Benjamin tenOever
Paul Tesar
Lenny Teytelman
Jamie Theobald
Michel Thiebaut de Schotten
Denis Thieffry
Lemberger Thomas
Véronique Thomas-Vaslin
Kimberly Thompson
William Hedley Thompson
Peter Thorn
Carl Thummel
Gregor Thut
Ralph Tiedemann
Matthew Tirrell
David Tollervey
Erdal Toprak
Benjamin Torben-Nielsen
Victor Torres
Jordi Torres-Rosell
Jonathan Towner
Simon Townsend
Michael Travisano
Matthew Traxler
Alain Trembleau

Michael Trenell
Elizabeth Tricomi
Marco Tripodi
Miltos Tsiantis
Kenichi Tsuda
Mick Tuite
James Tumlinson
Gustavo Turecki
Michael Turelli
Ted Turlings
John Tuthill
Mary Tyler
Mladen Tzvetkov
Naoyuki Uchida
Hiroki Ueda
Robert Unckless
David Unwin
Nathan Urban
Nathaniel Urban
Vladimir Uversky
Josef Uyeda
Robbie C. M. van Aert
Marcel van Assen
Josh Van Buskirk
Dedmer Van de Waal
Marcel van der Heijden
Rienk van Grondelle
Mark van Kleunen
Saskia van Mil
Monique van Oers
Virginie van Wassenhove
Josien van Wolfswinkel
Laura Vandenberg
Matthew Vander Heiden
Eric Vander Wal
Dieter Vanderelst
Wim Vanduffel
Rufin VanRullen
Julien Varaldi
Alexander Vargas
Harold Varmus
David Vaux
Jan-Willem Veening
Krishna Veeramah
Nicole Vega
Gregory Velicer
Kristen Verhey
Philippe Vernier
Simon Veron
Michael Verzi
Domenico Vicinanza
Pierre-Paul Vidal
K. VijayRaghavan
Devictor Vincent
Tim Vines
Mark Viney
Isaac Virshup
Peter Visscher
Edward Vogel
Martin Vogel
Claes von Wachenfeldt
Michiel Vos
Simone Vossel
Bradley Voytek
Vladyslav Vyazovskiy
Jill Waalen
Matt Wachowiak
Caroline S. Wagner
Henning Walczak
David Walker
Joseph Walston
Dagmar Waltemath
Yu Jui Wan
Julia Wang
Peng Wang
Zhanyun Wang
Jing Wang
Meng Wang
Rui Wang-Sattler
Jonathan Warner
Martin Warren
Claudia Waskow
Friedemann Weber
David Wedge
Bill Weis
Bernard Weissman
Nathan Weisz
Matthew Welch

Christine Wells
Dominic Wells
Nan-Ping Weng
Wolfgang Weninger
Thomas Wernberg
Sarah West
Stu West
Craig Wheelock
Patrick Whelan
Geoffrey While
Benjamin White
Richard White
Stephen White
Timothy Whitehead
George Whitesides
Malcolm Whitman
David Whitney
Robin Whyatt
Jelte Wicherts
Claude Wicker-Thomas
Lutz Wiegrebe
David Wiest
Gagan Wig
Eric Wilberg
Steven Wiley
Chris Willberg
Cranos Williams
Mark Williamson
Matthias Wilmanns
Christopher Wilmers
Rachel Wilson
Patricia Wittkopp
Benjamin Wolfe
Matthew Wolfgang
Michael Wolfgang
Mariana Wolfner
Jonathan Wolpaw
Lynn Wong
Sunny Wong
Kevin Wood
Shona Wood
Geoffrey Woodman
Robert Woods
Kevin Woollard
Boris Worm
Jun Wu
Keqiang Wu
Jinhua Wu
Hanno Wuerbel
Claire Wyart
Zhiyong Xi
Can Xie
Wei Xie
Na Xiong
Baoji Xu
Shawn Xu
Tal Yarkoni
Andrew Yates
Kuo-Chen Yeh
Joanne Yew
Calvin Yip
Kevin Young
Nathan Young
Hongtao Yu
Zhi-Min Yuan
Gabriel Yvon-Durocher
Deborah Zarin
Robert Zatorre
Daniel Zenklusen
Qing Zhang
Xiaoping Zhong
Daohong Zhou
Jinfang Zhu
Min Zhuo
Johannes Ziegler
Karl Zilles
Elana Zion Golumbic
Chenghang Zong
Wei-Xing Zong
Wei Zou
J. C. Zuniga-Pflucker
Mark Zwart
Joel Zylberberg
